# Clinical, para-clincal and outcomes of diabetes ketoacidosis in Vietnam national hospital pediatrics

**DOI:** 10.1186/1687-9856-2015-S1-P13

**Published:** 2015-04-28

**Authors:** Nguyen Thi Thu Nga, Nguyen Phu Dat, Vu Chi Dung, Bui Phuong Thao

**Affiliations:** 1 Hanoi Medical University, Hanoi, Vietnam; 2 Vietnam National Hospital of Pediatrics, Hanoi, Vietnam

**Keywords:** Diabetes ketoacidosis, DKA.

## 

Diabetic ketoacidosis (DKA) is a serious complication of diabetes mellitus that occurs when your body produces high levels of blood acids called ketones**.**

## Objective

This study aimed at describing some characteristics of clinical and commenting outcomes of the treatment for DKA in children with diabetes mellitus type 1 (DM1) in Vietnam National Hospital of Pediatrics from 2007 to 2012.

## Methods

Description retrospective and prospective on all patients previously diagnosed of DM1 or newly diagnosed of DM1 with DKA.

## Results

On average: each year, patients with DKA were admitted. 72% of these cases were first presentation with diabetes. Up to 36% of cases had misdiagnosis for other diseases. Clinical: dehydration 100%; 92% altered consciousness; vomiting 72%; thirsty 72%; weight loss 78%. The clinical presentation of DKA in patients who the newly diagnosed of DM1 was more severe than that in preciously diagnosed patients. The risk factors: 64% infection for all; 57.1% non-compliance with treatment for patients being treated insulin before. Laboratory: Blood glucose 36.8 ± 13.5 mol/l (from 14 to70 mol/l). Severe and moderate acidosis was seen in 56%. Abnormality of sodium levels were found in 11/25 patients (44%), of which only 3/25 patients actually decreased serum sodium (adjusted sodium); hyperkalemia in 6/25 patients (24%). The time of infusion insulin was 18.9 ± 27.56 hours. Average time replacing fluid was 37.7±21.1 hours. The duration of ketonuria was 37.9 ± 17.5 hours. Patients were alert after 12.04 ± 11.52 hours.

## Conclusion

DKA is a complex disordered metabolic state characterized by hyperglycemia, ketoacidosis, and ketonuria. The goal of treatment is to correct the high blood glucose level with insulin. Another goal is to replace fluids lost through urination, loss of appetite, and vomiting.

